# Electrochemical Aptasensor Based on rGO@gold Nanoparticles for Neuropeptide Y Detection

**DOI:** 10.3390/bios16070363

**Published:** 2026-07-02

**Authors:** Bin Gu, Weilong Tu, Biao Zou, Yuxian Chen, Qiaolin Fan, Cong Zhang, Xiao Li, Tao Hu

**Affiliations:** 1 School of Medicine, Southeast University, Nanjing 210009, China; gubiiin@163.com; 2 Jiangsu Key Laboratory for Design and Manufacturing of Precision Medicine Equipment, School of Mechanical Engineering, Southeast University, Nanjing 211189, Chinalx2016@seu.edu.cn (X.L.); 3 School of Mechanical and Electrical Engineering, Wuhan Institute of Technology, Wuhan 430205, China

**Keywords:** rGO, gold nanoparticles, electrochemical aptasensor, neuropeptide Y, reverse iontophoresis, wearable sensing

## Abstract

Neuropeptide Y (NPY) is a stress-modulating neuropeptide and a promising biomarker for non-invasive assessment. Herein, a sensitive electrochemical aptasensor was developed on reduced graphene oxide/gold nanoparticle (rGO/AuNP)-modified screen-printed electrodes for selective NPY detection. A methylene blue (MB)-labeled NPY-specific aptamer was immobilized on the electrode surface through Au–S chemistry, and square-wave voltammetry (SWV) was used for signal readout. The rGO/AuNP-modified interface provided high conductivity and a large effective surface area, facilitating electron transfer and probe immobilization. Under optimized conditions, the aptasensor exhibited a linear detection range of 10–10,000 pg mL^−1^ in PBS with a low detection limit of 1.17 pg mL^−1^ and good linearity (R^2^ = 0.991). In addition, the sensor showed satisfactory selectivity, reproducibility, and mechanical stability. Recovery tests in artificial sweat yielded recoveries of 91.8–107.8% with relative standard deviations below 5%, demonstrating good analytical accuracy in complex matrices. Combined with an agarose-hydrogel-assisted sampling interface and a reverse-iontophoresis-compatible wearable platform, this low-cost and facile sensing strategy provides a portable proof-of-concept approach for NPY analysis in artificial sweat and shows potential for future wearable-oriented biofluid monitoring.

## 1. Introduction

Neuropeptide Y (NPY) is a 36-amino acid peptide neurotransmitter that plays a crucial role in regulating multiple physiological processes, including appetite control, energy homeostasis, stress response, circadian rhythm, and cardiovascular regulation [1,2]. Abnormal expression levels of NPY have been closely associated with various pathological conditions, such as obesity, hypertension, metabolic syndrome, depression, and neurodegenerative diseases [3,4]. Therefore, accurate and sensitive detection of NPY is of great significance for both basic biomedical research and clinical diagnostics.

Traditional methods for NPY detection mainly include enzyme-linked immunosorbent assay (ELISA) [5], radioimmunoassay (RIA) [6], high-performance liquid chromatography (HPLC) [7], and mass spectrometry (MS) [8]. Although these techniques generally offer high sensitivity and specificity, they are often limited by complicated sample pretreatment, lengthy procedures, expensive instrumentation, and the requirement for trained personnel. These limitations restrict their implementation in point-of-care testing and wearable monitoring scenarios, highlighting the need for alternative analytical platforms that combine high sensitivity, rapid response, cost-effectiveness, and operational simplicity.

Electrochemical biosensing has emerged as a powerful approach for biomolecular detection owing to its high sensitivity, rapid signal transduction, portability, and suitability for miniaturization [9,10]. Among various electrode platforms, screen-printed electrodes (SPEs) have attracted widespread attention because of their low fabrication cost, ease of mass production, and compatibility with flexible and wearable devices [11,12,13]. The performance of SPE-based sensors can be significantly improved by integrating functional nanomaterials such as MXene, gold nanoparticles (AuNPs), and carbon-based nanostructures, which provide a large surface area, excellent conductivity, and abundant sites for probe immobilization [14,15,16,17,18]. Aptamers, which are single-stranded DNA or RNA oligonucleotides selected through systematic evolution of ligands by exponential enrichment (SELEX), have emerged as highly promising molecular recognition probes [19]. Compared with conventional antibodies, aptamers offer several advantages, including low-cost synthesis, excellent chemical stability, and facile modification [20,21,22]. These properties make them particularly suitable for integration into electrochemical sensing platforms [23]. In this study, a previously validated aptamer with high specificity toward NPY was employed [24,25,26]. Furthermore, methylene blue (MB) was introduced as an intrinsic electroactive label, enabling direct electrochemical readout without additional labeling procedures [27,28]. This strategy simplifies the sensing process while maintaining high analytical sensitivity and reliability. Collectively, these characteristics make electrochemical aptasensors particularly attractive for portable and wearable biofluid monitoring applications.

In recent years, wearable biochemical sensing technologies have attracted increasing attention owing to their potential for continuous and non-invasive health monitoring [29,30]. Among various biofluids, sweat has emerged as an attractive analytical medium because it can be collected painlessly and repeatedly without specialized personnel or invasive sampling procedures. Previous studies have identified NPY as a promising sweat-associated biomarker related to stress regulation and neurological conditions, and flexible sweat-based sensing platforms for NPY detection have been reported [31]. Reported physiological concentrations of NPY in sweat generally fall within the low-pg mL^−1^ to hundreds-pg mL^−1^ range, while previously reported sweat-based NPY sensing systems typically cover analytical ranges of approximately 1–256 pg mL^−1^. These concentration levels place stringent requirements on sensor sensitivity and analytical performance, highlighting the need for highly sensitive and wearable-compatible sensing platforms for non-invasive sweat NPY analysis.

**Scheme 1:**
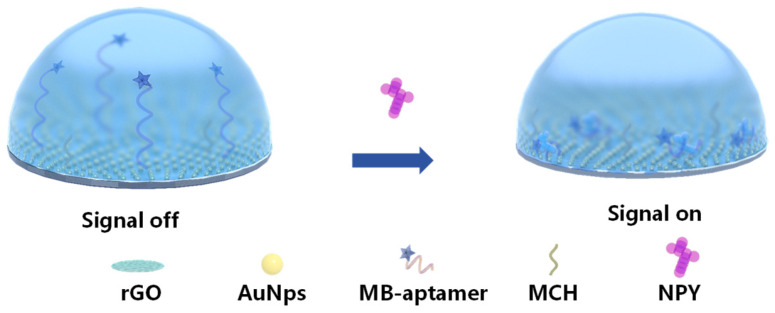
Schematic of electrochemical aptasensor principle based on rGO/AuNPs for NPY detection.

Although graphene/gold-based electrochemical NPY aptasensors have been reported previously, including the graphene–gold nanocomposite platform developed by Fernandez et al. for label-free NPY detection through dielectrophoretic enrichment, their application has primarily focused on buffer-based analytical systems rather than wearable biofluid monitoring [32,33,34,35,36]. Quantitative validation in sweat-related matrices and integration with wearable-compatible sampling configurations remain largely unexplored. To address these challenges, we developed an MB-labeled NPY aptamer sensor on rGO/AuNP-modified screen-printed electrodes (Scheme 1). The rGO/AuNP layer served as a conductive and high-surface-area immobilization interface, while the MB-labeled aptamer enabled direct square-wave voltammetry (SWV)-based signal readout. Furthermore, the sensing platform was integrated with an agarose hydrogel-assisted sampling interface and a reverse-iontophoresis-compatible wearable configuration. The developed platform was systematically evaluated in terms of sensitivity, selectivity, reproducibility, mechanical stability, and recovery performance in artificial sweat. Compared with previously reported graphene/gold-based NPY sensing systems, the present platform combines flexible screen-printed electrodes, artificial sweat validation, and wearable-compatible sampling integration, thereby extending NPY sensing toward portable and non-invasive biofluid monitoring applications. This integrated design provides a proof-of-concept platform for sweat-related NPY analysis and highlights the translational potential of wearable electrochemical sensing for future biofluid monitoring applications.

## 2. Materials and Methods

### 2.1. Materials and Apparatus

The Neuropeptide Y (NPY)-specific aptamer, purified by HPLC, was purchased from Sangyo Biotech (Shanghai, China). Its sequence was 5′-HS-SH C6-AGC AGC ACA GAG GTC AGA TGC AAA CCA CAG CCT GAG TGG TTA GCG TAT GTC ATT TAC GGA CCT ATG CGT GCT ACC GTG AA-MB-3′, with a methylene blue (MB) modification at the 3′ terminus completed by the supplier. Tris-HCl buffer (1 M, pH 8.0, sterile) was also obtained from Sangyo Biotech. NPY and tetrachloroauric acid (HAuCl_4_) were used directly. Artificial sweat (pH 7.4) was acquired from Yuan Ye (Shanghai, China). Additional chemicals included NaCl, MgCl_2_, FeCl_3_, K_3_[Fe(CN)_6_], K_4_[Fe(CN)_6_], Tris[2-carboxyethyl] phosphine (TCEP), ultrapure water, glucose (Glu), cortisol, BSA, graphene oxide dispersion (2 mg/mL), cysteine, agarose, and phosphate-buffered saline (PBS, 0.1 M, pH 7.4). All reagents were of analytical grade and used without further purification. Scanning electron microscopy (SEM, FEI Inspect F50, Thermo Fisher, Waltham, MA, USA) and electrochemical analyses were performed on the Autolab electrochemical workstation (Metrohm, Herisau, Switzerland).

### 2.2. Preparation of rGO/AuNP-Modified SPE

First, 2 mL of graphene oxide dispersion (2 mg mL^−1^) was placed in a clean glass vial and stirred at room temperature for 30 min. L-cysteine was dissolved in 2 mL of ultrapure water and sonicated in an ice bath for 30 min. To optimize the reduction condition, different mass ratios of L-cysteine to GO (1:1, 2:1, 4:1, and 8:1) were investigated. The cysteine solution was then slowly added to the GO dispersion and stirred at room temperature for 1 h. For the fabrication of the final sensor, the optimal mass ratio of 4:1 was used. Screen-printed electrodes (SPEs) were rinsed with ultrapure water, dried, and treated with plasma. Then, 5 μL of the GO/L-cysteine mixture was drop-cast onto the working electrode area. The electrodes were placed in a sealed container and incubated in a 70 °C water bath for 12 h to obtain SPE/rGO. To optimize AuNP deposition, different electrodeposition times (20, 30, 40, 50, and 60 s) were examined in 1.5 mg mL^−1^ HAuCl_4_ solution at −0.6 V. For the fabrication of the final sensor, the optimal deposition time of 50 s was used. After deposition, the electrodes were rinsed with ultrapure water and dried at 60 °C to obtain SPE/rGO/AuNPs.

### 2.3. Fabrication of NPY Aptasensor

The aptamer was reduced using tris (2-carboxyethyl) phosphine (TCEP). A 200 µL solution of 15 µM aptamer was mixed with 5 µL of 1 M TCEP in binding buffer (50 mM Tris-HCl, 137 mM NaCl, 5 mM MgCl_2_) and incubated at room temperature for 30 min. After dilution, 7 µL of the solution was dropped onto the electrode surface and incubated at 4 °C for 18 h. Unbound aptamers were removed by rinsing, and the electrode was dried under nitrogen. Subsequently, 5 µL of 1 mM β-mercaptoethanol (MCH) was added and left for 30 min to block residual active sites on the gold surface. The electrode was rinsed and nitrogen-dried to obtain the final SPE/rGO/AuNPs aptasensor.

### 2.4. NPY Detection with Aptasensor

All cyclic voltammetry (CV) and electrochemical impedance spectroscopy (EIS) measurements were carried out in a 5 mM [Fe(CN)_6_]^3−/4−^ solution containing 0.1 M KCl as the supporting electrolyte. PBS and artificial sweat solutions with varying NPY concentrations were directly applied to the working electrode (10 μL). Square-wave voltammetry (SWV) measurements were performed over a potential range from −0.6 V to 0.2 V (vs. Ag/AgCl) with a step potential of 5 mV, a modulation amplitude of 0.1 mV, a frequency of 5 Hz, an interval time of 0.2 s, and a scan rate of 0.025 V s^−1^. ΔI represents the change in current, where ΔI = I − I_0_, I is the peak SWV current under test conditions and I_0_ is the peak SWV current in the absence of NPY. The limit of detection (LOD) was calculated from blank measurements according to the 3σ criterion. Specifically, the threshold signal was defined as ΔI_LOD_ = ΔI_blank_ + 3.3σ_blank_, where σ_blank_ is the standard deviation of repeated blank measurements (*n* = 3). The corresponding NPY concentration was then obtained by substituting ΔI_LOD_ into the logarithmic calibration equation.

## 3. Results and Discussion

**Figure 1:**
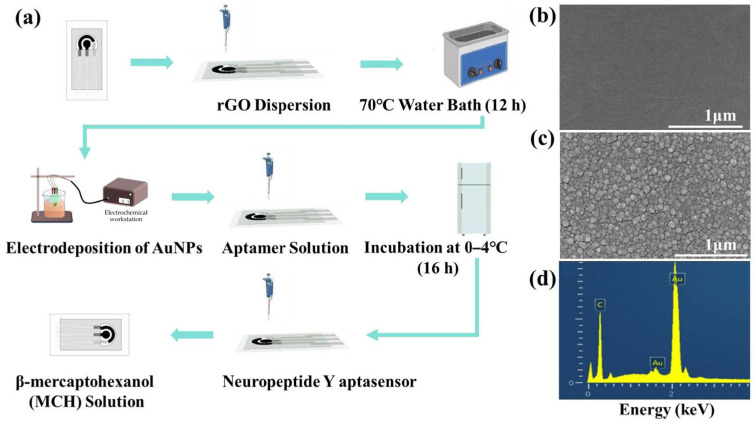
(**a**) Schematic illustration of the fabrication procedure of the rGO/AuNP-based electrochemical aptasensor for NPY detection. (**b**) SEM image of the rGO-modified screen-printed electrode. (**c**) SEM image of AuNPs electrodeposited on the rGO-modified electrode. (**d**) Corresponding EDS spectrum of the rGO/AuNP-modified electrode.

Figure 1a illustrates the fabrication procedure of the rGO/AuNP-based electrochemical aptasensor for NPY detection. The preparation process involves the drop-casting of rGO onto the screen-printed electrode (SPE), followed by electrodeposition of Au nanoparticles (AuNPs), immobilization of the methylene blue-labeled aptamer, and subsequent blocking with β-mercaptohexanol (MCH) to obtain the final NPY aptasensor. The surface morphology of the modified electrodes was characterized by scanning electron microscopy (SEM). As shown in Figure 1b, the rGO-modified SPE exhibits a relatively uniform and smooth surface morphology, indicating that rGO forms a continuous coating on the electrode surface, which provides a conductive substrate for subsequent AuNP deposition. After electrodeposition, densely distributed AuNPs with a well-defined spherical morphology are observed on the rGO surface (Figure 1c), confirming the successful formation of the nanostructured Au layer. The corresponding energy-dispersive X-ray spectroscopy (EDS) spectrum (Figure 1d) displays characteristic peaks of Au and C. The C signal originates from both rGO and the conductive carbon ink substrate of the SPE, while the pronounced Au signal further verifies the successful deposition of AuNPs on the electrode surface.

**Figure 2:**
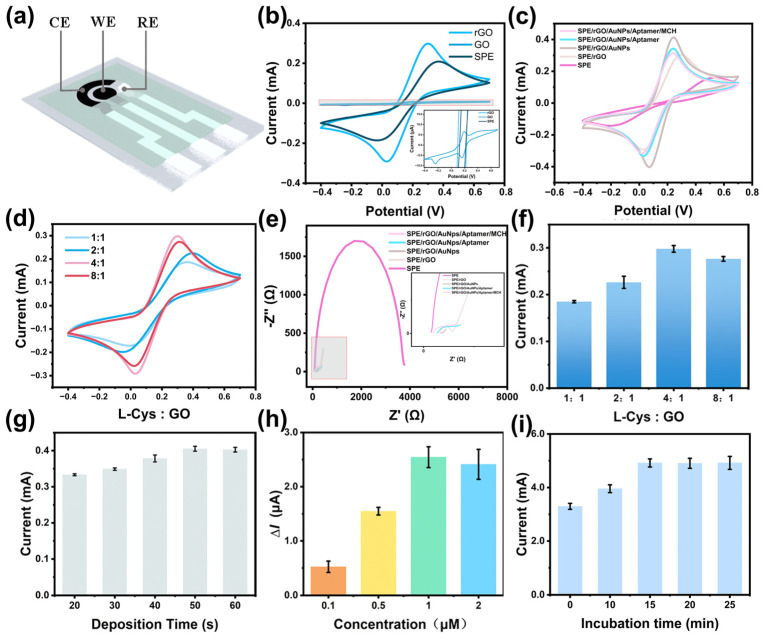
(**a**) Schematic diagram of the screen-printed electrode (SPE) showing CE, WE, and RE. (**b**) CV curves of SPE, SPE/GO, and SPE/rGO electrodes. (**c**) CV curves of stepwise modified electrodes (SPE, SPE/rGO, SPE/rGO/AuNPs, SPE/rGO/AuNPs/aptamer, and SPE/rGO/AuNPs/aptamer/MCH). (**d**–**f**) Optimization of the mass ratio of L-cysteine to GO. (**g**) Effect of AuNPs’ electrodeposition time on the electrochemical response. (**h**) Optimization of aptamer concentration. (**i**) Effect of incubation time on the sensing response.

Electrochemical measurements were further carried out to evaluate the electrode modification process. Figure 2a shows a schematic diagram of the screen-printed electrode (SPE), including the counter electrode (CE), working electrode (WE), and reference electrode (RE). To verify the successful reduction of GO, cyclic voltammetry (CV) measurements were performed in a 5 mM [Fe(CN)_6_]^3−/4−^ solution containing 0.1 M KCl over a potential range of −0.4 to 0.7 V at a scan rate of 50 mV s^−1^ using bare SPE, SPE/GO, and SPE/rGO electrodes. As shown in Figure 2b, GO modification leads to a noticeable decrease in the redox peak currents due to its relatively low conductivity. In contrast, SPE/rGO exhibits significantly enhanced peak currents, indicating that GO was effectively reduced to rGO. To further monitor each modification step, cyclic voltammetry (CV) and electrochemical impedance spectroscopy (EIS) measurements were conducted for SPE, SPE/rGO, SPE/rGO/AuNPs, SPE/rGO/AuNPs/Aptamer, and SPE/rGO/AuNPs/Aptamer/MCH electrodes. As shown in Figure 2c,e, the EIS spectra were further fitted using an equivalent circuit model to quantitatively evaluate the interfacial electron-transfer properties (Figure S3, Supplementary Materials). The corresponding Rct values (Table S1, Supplementary Materials) were determined to be 3584.0 Ω, 110.2 Ω, 22.6 Ω, 240.7 Ω, and 219.4 Ω for SPE, SPE/rGO, SPE/rGO/AuNPs, SPE/rGO/AuNPs/Aptamer, and SPE/rGO/AuNPs/Aptamer/MCH, respectively. The dramatic decrease in R_ct_ after rGO modification and subsequent AuNP deposition, together with the enhanced CV peak currents, confirms the significant improvement in electrode conductivity and electron-transfer kinetics. This enhancement can be attributed to the excellent conductivity of rGO and the additional conductive pathways and high-surface-area interface provided by AuNPs. Following aptamer immobilization and MCH blocking, the CV peak currents decreased while Rct increased markedly, owing to the formation of insulating biomolecular layers on the electrode surface. These results further verify the successful stepwise assembly of the sensing interface.

To further improve the electrochemical performance of the sensor, the mass ratio of L-cysteine to graphene oxide was optimized. As shown in Figure 2d,f, the oxidation peak current gradually increases with increasing L-cysteine content and reaches a maximum at a ratio of 4:1. When the ratio is further increased to 8:1, the current slightly decreases, which may be attributed to excessive cysteine coverage that hinders electron transfer. Therefore, a ratio of 4:1 was selected as the optimal condition for subsequent experiments. The electrodeposition time of AuNPs was also optimized to achieve the best electrochemical performance. As shown in Figure 2g, the oxidation peak current increases with increasing deposition time from 20 to 50 s, indicating improved conductivity and effective formation of AuNPs. When the deposition time is extended to 60 s, the current response shows only a slight increase, suggesting that excessive deposition provides limited additional benefit. Therefore, 50 s was selected as the optimal electrodeposition time.

Furthermore, the aptamer concentration was optimized to obtain the best sensing response toward NPY. As illustrated in Figure 2h, the signal change (ΔI) increases with increasing aptamer concentration and reaches a maximum at 1 μM, suggesting that an appropriate surface coverage of aptamer molecules was achieved at this concentration. Therefore, 1 μM was selected as the optimal aptamer concentration. Finally, the incubation time between the aptamer-modified electrode and NPY was optimized. As shown in Figure 2i, the sensing response increases with incubation time and reaches a plateau at approximately 15 min, indicating that the binding between the aptamer and NPY approaches equilibrium. Therefore, an incubation time of 15 min was selected for subsequent experiments.

**Figure 3:**
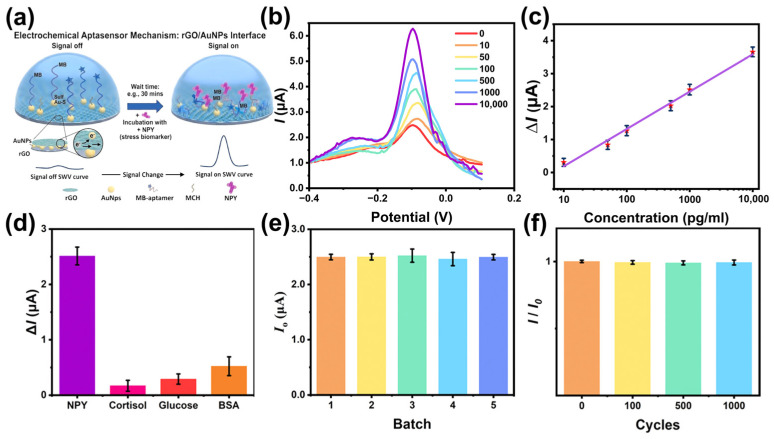
(**a**) Sensing mechanism of the rGO/AuNP-based electrochemical aptasensor for NPY detection. (**b**) SWV responses of the aptasensor to increasing NPY concentrations (0–10,000 pg mL^−1^). (**c**) Calibration curve of ΔI versus NPY concentration. (**d**) Selectivity of the aptasensor toward 1 ng mL^−1^ NPY against 100 ng mL^−1^ potential interferents, including cortisol, glucose, and BSA. (**e**) Reproducibility of the aptasensor evaluated from five independently prepared electrodes. (**f**) Stability of the aptasensor after repeated bending cycles.

Figure 3a illustrates the sensing mechanism of the rGO/AuNP-based electrochemical aptasensor for NPY detection. Upon binding of NPY to the methylene blue (MB)-labeled aptamer immobilized on the electrode surface, the interfacial electron-transfer behavior is altered, resulting in a measurable change in the square-wave voltammetry (SWV) signal. The concentration-dependent response of the aptasensor toward NPY in PBS is shown in Figure 3b. As the NPY concentration increases from 0 to 10,000 pg mL^−1^, the SWV peak current gradually increases, indicating enhanced interactions between NPY and the MB-labeled aptamer. This binding process influences the spatial orientation of the MB tag and thereby modulates the electron-transfer efficiency between MB and the electrode surface. As shown in Figure 3c, a clear linear relationship is observed between ΔI and NPY concentration within the tested range. The regression equation can be expressed as ΔI_μA_ = (1.133 ± 0.049) log(C_NPY_) [×10–12 g mL^−1^] − (0.946 ± 0.121), with a correlation coefficient (R^2^) of 0.991 (*n* = 3). The sensitivity of the sensor was calculated to be 9.25 μA (lg[pg mL^−1^])^−1^ cm^−2^. Based on repeated blank measurements and the 3σ criterion, the LOD was calculated to be 1.17 pg mL^−1^ by converting the threshold current response through the logarithmic calibration equation. These results demonstrate that the aptasensor provides a stable and concentration-dependent electrochemical response toward NPY.

The selectivity of the electrochemical aptasensor was evaluated using cortisol, glucose, and bovine serum albumin (BSA) as potential interferents, representing endogenous small molecules, metabolites, and macromolecular proteins, respectively. As shown in Figure 3d, under the conditions of 1 ng mL^−1^ NPY and 100 ng mL^−1^ interferents, the ΔI response toward NPY is significantly higher than that toward cortisol, glucose, and BSA, with all interference signals remaining below 0.7 μA. The non-overlapping error bars further confirm the statistical difference, indicating good selectivity of the sensor toward NPY. The inter-electrode reproducibility of the aptasensor was assessed using five independently prepared electrodes from the same fabrication batch. As shown in Figure 3e, the initial peak currents (I_0_) of the five electrodes are highly consistent, demonstrating good within-batch fabrication reproducibility. Batch-to-batch reproducibility was not systematically evaluated in this study and will be further investigated in future scale-up fabrication. To evaluate the mechanical durability of the sensor, cyclic bending tests were performed, in which one bending cycle was defined as bending the device to 90° and then returning it to a flat state. The electrochemical response toward 1000 pg mL^−1^ NPY was recorded after 0, 100, 500, and 1000 bending cycles. As shown in Figure 3f, the SWV peak current ratio (I/I_0_) remains close to 1.0 without obvious decay, indicating that the rGO/AuNP-modified flexible electrode maintains stable interfacial integrity and electrochemical performance after repeated mechanical deformation.

**Figure 4:**
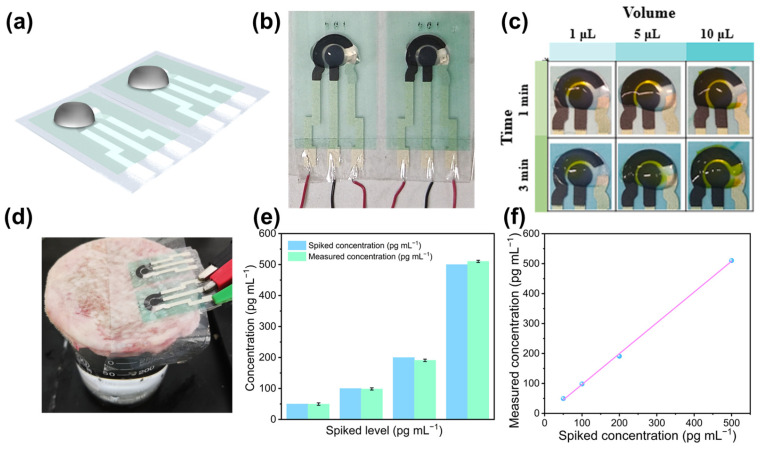
(**a**) Schematic illustration of the reverse-iontophoresis-compatible wearable electrochemical aptasensor platform for sweat-related sampling and NPY detection. (**b**) Photograph of the fabricated wearable-compatible sensor. (**c**) Diffusion behavior of droplets on the agarose hydrogel-modified electrode surface at different volumes (1, 5, and 10 μL) and times (1 and 3 min), evaluated using orange food dye to determine the optimal sample volume for NPY detection. (**d**) Photograph demonstrating the wearable sensor during the artificial sweat extraction experiment. (**e**) Recovery performance of the aptasensor for NPY detection in artificial sweat at different spiked concentrations (50–500 pg mL^−1^). (**f**) Linear correlation between spiked and measured NPY concentrations in artificial sweat.

To explore the wearable compatibility of the aptasensor in sweat-related scenarios, a reverse-iontophoresis-compatible sensing platform was further constructed as a proof-of-concept device configuration. As shown in Figure 4a, the wearable device integrates the electrochemical sensing unit with an electrically assisted sampling module, indicating the potential to combine sweat-related sampling and electrochemical detection in a single platform. It should be noted that, in the present study, quantitative NPY detection was validated using spiked artificial sweat rather than NPY extracted through human skin or a skin-mimicking membrane. Figure 4b presents a photograph of the fabricated sensor, demonstrating its flexible structure and wearable-compatible configuration. To improve sweat collection and maintain interfacial stability, an agarose hydrogel layer was introduced onto the sensing area of the flexible electrode. The hydrogel-modified sensor was further evaluated by diffusion experiments using orange food dye at different loading volumes (1, 5, and 10 μL) and standing times (1 and 3 min). As shown in Figure 4c, the 10 μL droplet exhibits the most uniform distribution within the hydrogel layer after 3 min, indicating sufficient liquid spreading and effective coverage of the sensing area. This result suggests that 10 μL is an appropriate sample volume for subsequent NPY detection and also demonstrates that the hydrogel-modified sensor can operate with only a small amount of test solution. Figure 4d shows the wearable sensor during the artificial sweat extraction experiment, further demonstrating the feasibility of the integrated device for practical operation. To verify the analytical accuracy of the aptasensor in complex matrices, recovery experiments were performed in artificial sweat by spiking NPY standard solutions at concentrations ranging from 50 to 500 pg mL^−1^. As shown in Figure 4e, the measured concentrations are in good agreement with the corresponding spiked values, indicating satisfactory recovery performance. The detailed recovery results are summarized in Table 1. The recoveries range from 91.8% to 107.8%, with relative standard deviations (RSDs) below 5%, confirming the high analytical accuracy of the developed aptasensor in artificial sweat. Furthermore, the relationship between the spiked and measured NPY concentrations is shown in Figure 4f, where a good linear correlation is obtained. These results confirm that the developed electrochemical aptasensor exhibits reliable quantitative performance in artificial sweat and supports its potential for sweat-related wearable analysis. However, several challenges remain before practical on-body NPY monitoring can be achieved. First, sweat secretion rate, pH, ionic strength, and biomolecular composition vary substantially among individuals and physiological states, which may affect aptamer accessibility and electrochemical signal stability. Second, skin deformation and body motion during long-term wear may disturb the hydrogel–electrode–skin interface and lead to unstable sampling or signal drift. Third, the agarose hydrogel layer must maintain sufficient hydration and anti-fouling performance during prolonged operation. In addition, reverse iontophoresis parameters should be optimized to balance sampling efficiency, skin comfort, and electrical safety. Therefore, future work will focus on real-sweat validation, skin-model extraction studies, long-term on-body stability assessment, and integration with miniaturized electronics to enable continuous wearable monitoring.

## 4. Conclusions

In summary, a flexible electrochemical aptasensor based on rGO/AuNP-modified screen-printed electrodes was successfully developed for sensitive NPY detection. The incorporation of rGO/AuNPs improved the interfacial conductivity and facilitated probe immobilization, while the MB-labeled aptamer provided selective molecular recognition and direct electrochemical signal transduction. Under optimized conditions, the aptasensor exhibited a wide linear range of 10–10,000 pg mL^−1^, a low detection limit of 1.17 pg mL^−1^, and satisfactory selectivity, reproducibility, and mechanical stability. Recovery experiments in artificial sweat further confirmed its good analytical accuracy in complex matrices. Moreover, the integration of the sensing unit with an agarose hydrogel-assisted sampling interface and a reverse-iontophoresis-compatible wearable configuration demonstrated the feasibility of device-level integration for future sweat-related wearable applications. These results suggest that the proposed platform provides a promising approach for portable NPY analysis and may serve as a useful framework for the development of wearable electrochemical sensors targeting other non-invasive biomarkers. Future studies will focus on validation in real sweat samples and further optimization of the integrated wearable platform under physiological conditions.

## Figures and Tables

**Table 1:** NPY concentration measured in artificial sweat (*n* = 3).

Sample	Spiked Concentration (pg mL^−1^)	Measured Concentration (pg mL^−1^) ± SD	Recovery (%)	RSD (%)
1	50	47.7 ± 1.9	95.4	4.0
2	100	102.3 ± 3.8	102.3	3.7
3	200	183.6 ± 7.2	91.8	3.9
4	500	529.2 ± 18.8	107.8	3.5

## Data Availability

The data presented in this study are available on request from the corresponding author.

## Supplementary Materials

The following supporting information can be downloaded at: https://www.mdpi.com/article/10.3390/bios16070363/s1. Figure S1: Schematic illustration of the operating principle of the semi-automatic screen-printing machine (SPC-3050), showing the squeegee, screen-printing mesh plate, and PET film during the printing process; Figure S2: Schematic layout of the screen-printing stencil patterns for the fabrication of the base electrodes; Figure S3: Equivalent circuit model employed for fitting the electrochemical impedance spectroscopy (EIS) spectra and comparison between experimental EIS spectra and fitted results for SPE, SPE/rGO, SPE/rGO/AuNPs, SPE/rGO/AuNPs/Aptamer, and SPE/rGO/AuNPs/Aptamer/MCH electrodes; Table S1: Charge-transfer resistance (R_ct_) values obtained from equivalent-circuit fitting of the EIS spectra for different electrodes.

## References

[1] Heilig M. (2004). The NPY system in stress, anxiety and depression. Neuropeptides.

[2] Eaton K., Sallee F.R., Sah R. (2007). Relevance of neuropeptide Y (NPY) in psychiatry. Curr. Top. Med. Chem..

[3] Reichmann F., Holzer P. (2016). Neuropeptide Y: A stressful review. Neuropeptides.

[4] Lu J., Li S., Li H., Mou T., Zhou L., Huang B., Huang M., Xu Y. (2019). Changes in plasma NPY, IL-1β and hypocretin in people who died by suicide. Neuropsychiatr. Dis. Treat..

[5] Jia M., Belyavskaya E., Deuster P., Sternberg E.M. (2012). Development of a sensitive microarray immunoassay for the quantitative analysis of neuropeptide Y. Anal. Chem..

[6] Theodorsson-Norheim E., Hemsén A., Lundberg J.M. (1985). Radioimmunoassay for neuropeptide Y (NPY): Chromatographic characterization of immunoreactivity in plasma and tissue extracts. Scand. J. Clin. Lab. Investig..

[7] Salsen L., Andersen H., Bratholm P., Christensen N. (1994). Radioimmunoassay of plasma neuropeptide Y using HPLC for separation of related peptides and fragments. Scand. J. Clin. Lab. Investig..

[8] Vocat C., Dunand M., Hubers S.A., Bourdillon N., Millet G.P., Brown N.J., Wuerzner G., Grouzmann E., Eugster P.J. (2019). Quantification of neuropeptide Y and four of its metabolites in human plasma by micro-UHPLC-MS/MS. Anal. Chem..

[9] Ronkainen N.J., Halsall H.B., Heineman W.R. (2010). Electrochemical biosensors. Chem. Soc. Rev..

[10] Wu J., Liu H., Chen W., Ma B., Ju H. (2023). Device integration of electrochemical biosensors. Nat. Rev. Bioeng..

[11] Arduini F., Micheli L., Moscone D., Palleschi G., Piermarini S., Ricci F., Volpe G. (2016). Electrochemical biosensors based on nanomodified screen-printed electrodes: Recent applications in clinical analysis. TrAC Trends Anal. Chem..

[12] Singh A., Sharma A., Ahmed A., Sundramoorthy A.K., Furukawa H., Arya S., Khosla A. (2021). Recent advances in electrochemical biosensors: Applications, challenges, and future scope. Biosensors.

[13] Mincu N.-B., Lazar V., Stan D., Mihailescu C.M., Iosub R., Mateescu A.L. (2020). Screen-Printed Electrodes (SPE) for in vitro diagnostic purpose. Diagnostics.

[14] Gao Y., Wu X., Wang H., Lu W., Guo M. (2018). Highly sensitive detection of hesperidin using AuNPs/rGO modified glassy carbon electrode. Analyst.

[15] Xu B., Zhi C., Shi P. (2020). Latest advances in MXene biosensors. J. Phys. Mater..

[16] Devadoss A., Han H., Song T., Kim Y.-P., Paik U. (2013). Gold nanoparticle-composite nanofibers for enzymatic electrochemical sensing of hydrogen peroxide. Analyst.

[17] Krishnan S.K., Singh E., Singh P., Meyyappan M., Nalwa H.S. (2019). A review on graphene-based nanocomposites for electrochemical and fluorescent biosensors. RSC Adv..

[18] Wei J., Qiu J., Li L., Ren L., Zhang X., Chaudhuri J., Wang S. (2012). A reduced graphene oxide based electrochemical biosensor for tyrosine detection. Nanotechnology.

[19] Cheng A.K., Sen D., Yu H.-Z. (2009). Design and testing of aptamer-based electrochemical biosensors for proteins and small molecules. Bioelectrochemistry.

[20] He L., Huang R., Xiao P., Liu Y., Jin L., Liu H., Li S., Deng Y., Chen Z., Li Z. (2021). Current signal amplification strategies in aptamer-based electrochemical biosensor: A review. Chin. Chem. Lett..

[21] Sangili A., Kalyani T., Chen S.-M., Nanda A., Jana S.K. (2020). Label-free electrochemical immunosensor based on one-step electrochemical deposition of AuNP-RGO nanocomposites for detection of endometriosis marker CA 125. ACS Appl. Bio Mater..

[22] Zor E., Bingol H., Ramanaviciene A., Ramanavicius A., Ersoz M. (2015). An electrochemical and computational study for discrimination of d- and l-cystine by reduced graphene oxide/β-cyclodextrin. Analyst.

[23] Ge S., Yan M., Lu J., Zhang M., Yu F., Yu J., Song X., Yu S. (2012). Electrochemical biosensor based on graphene oxide–Au nanoclusters composites for l-cysteine analysis. Biosens. Bioelectron..

[24] Tabrizi M.A., Shamsipur M., Saber R., Sarkar S., Besharati M. (2018). An electrochemical aptamer-based assay for femtomolar determination of insulin using a screen printed electrode modified with mesoporous carbon and 1,3,6,8-pyrenetetrasulfonate. Microchim. Acta.

[25] Du Y., Li B., Wei H., Wang Y., Wang E. (2008). Multifunctional label-free electrochemical biosensor based on an integrated aptamer. Anal. Chem..

[26] Shao Y., Wang J., Wu H., Liu J., Aksay I.A., Lin Y. (2010). Graphene based electrochemical sensors and biosensors: A review. Electroanalysis.

[27] Dan Y., Lu Y., Kybert N.J., Luo Z., Johnson A.C. (2009). Intrinsic response of graphene vapor sensors. Nano Lett..

[28] Wang Y., Jie H., Ye H., Zhang Y., Li N., Zhuang J. (2023). Methylene blue-stained single-stranded DNA aptamers as a highly efficient electronic switch for quasi-reagentless exosomes detection: An old dog with new tricks. Anal. Chem..

[29] Gao F., Liu C., Zhang L., Liu T., Wang Z., Song Z., Xue N. (2023). Wearable and flexible electrochemical sensors for sweat analysis: A review. Microsyst. Nanoeng..

[30] Min J., Tu J., Xu C., Lukas H., Shin S., Yang Y., Gao W. (2023). Skin-interfaced wearable sweat sensors for precision medicine. Chem. Rev..

[31] Churcher N.K.M., Upasham S., Rice P., Bhadsavle S., Prasad S. (2020). Development of a flexible, sweat-based neuropeptide Y detection platform. RSC Adv..

[32] Lopez L., Hernandez N., Morales J.R., Cruz J., Flores K., Gonzalez-Amoretti J., Rivera V., Cunci L. (2021). Measurement of Neuropeptide Y Using Aptamer-Modified Microelectrodes by Electrochemical Impedance Spectroscopy. Anal. Chem..

[33] Lopez L., Martinez L.M., Caicedo J.R., Fernandez-Vega L., Cunci L. (2024). Measurement of Neuropeptide Y in Aptamer-Modified Planar Electrodes. Electrochim. Acta.

[34] Richardson H., Maddocks G., Peterson K., Daniele M., Pavlidis S. (2021). Toward Subcutaneous Electrochemical Aptasensors for Neuropeptide Y. 2021 IEEE Sensors.

[35] Fernandez R.E., Sanghavi B.J., Farmehini V., Chávez J.L., Hagen J., Kelley-Loughnane N., Chou C.-F., Swami N.S. (2016). Aptamer-functionalized graphene-gold nanocomposites for label-free detection of dielectrophoretic-enriched neuropeptide Y. Electrochem. Commun..

[36] Seibold J.M., Abeykoon S.W., Ross A.E., White R.J. (2023). Development of an Electrochemical, Aptamer-Based Sensor for Dynamic Detection of Neuropeptide Y. ACS Sens..

