# Supplementary material for: Electrochemical Aptasensor Based on rGO@gold Nanoparticles for Neuropeptide Y Detection
Source: Biosensors (Basel). 2026 Jul 2;16(7):363. doi: 10.3390/bios16070363 (PMC13406597; doi:10.3390/bios16070363)
Supplement: Supplementary file 1 [file biosensors-16-00363-s001.zip › biosensors-4307605-supplementary.pdf]

Supporting Information

Electrochemical Aptasensor Based on rGO@gold Nanoparticles for Neuropeptide Y Detection

Bin Gu,[1] Weilong Tu,[2] Biao Zou,[2] Yuxian Chen,[2] Qiaolin Fan,[2] Cong Zhang,[2,3] and Tao Hu[2*]*

[1] School of Medicine, Southeast University, Nanjing, Jiangsu Province, China, 210009

[2] School of Mechanical Engineering, Jiangsu Key Laboratory for Design and Manufacturing of Precision Medicine Equipment, Southeast University, Nanjing, Jiangsu Province, China, 211189

[3] School of Mechanical and Electrical Engineering, Wuhan Institute of Technology, Wuhan, China, 430205

Corresponding Author

*E-mail: hutao@seu.edu.cn (Tao Hu), zhangcong94@foxmail.com(C. Zhang)

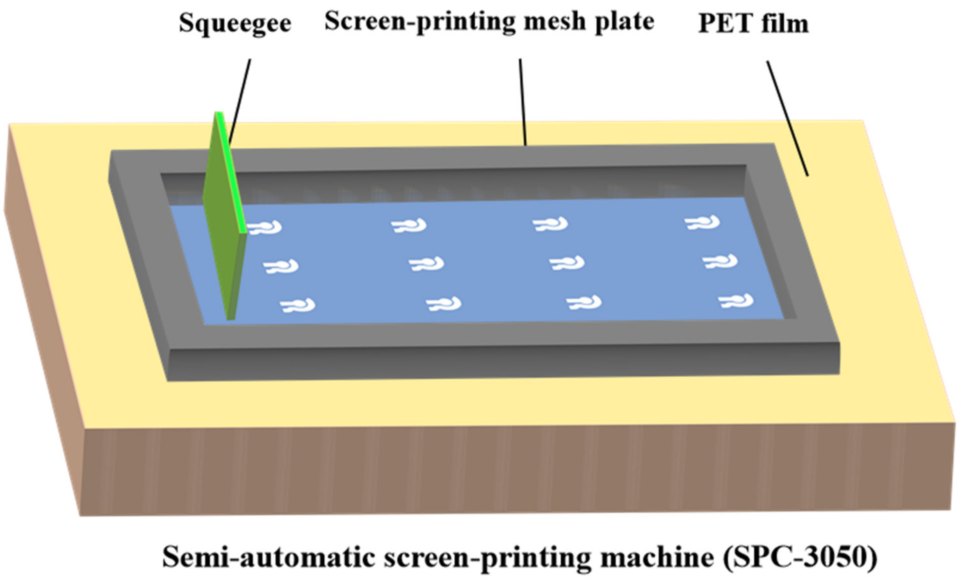

Figure S1. Schematic illustration of the operating principle of the semi-automatic screen-printing machine (SPC-3050), showing the squeegee, screen-printing mesh plate, and PET film during the printing process.

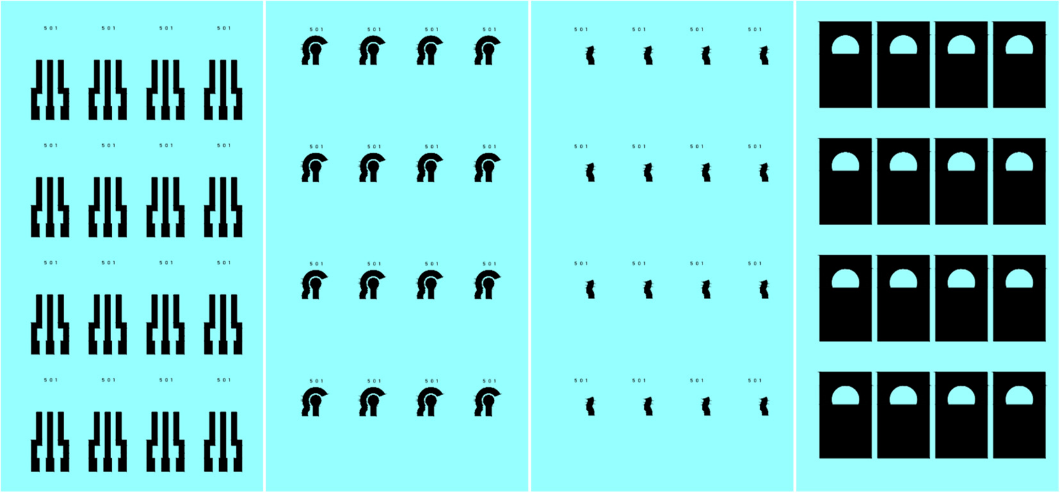

Figure S2. Schematic layout of the screen-printing stencil patterns for the fabrication of the base electrodes.

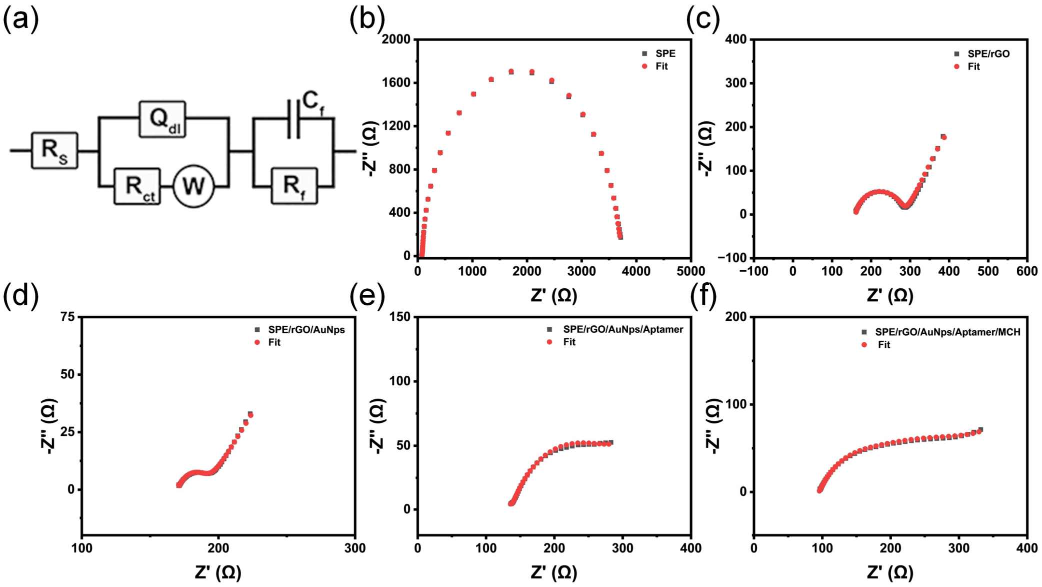

Figure S3. (a) Equivalent circuit model employed for fitting the electrochemical impedance spectroscopy (EIS) spectra. R_s denotes the solution resistance, R_{ct} the charge-transfer resistance, Qdl the constant phase element of the electrical double layer, W the Warburg diffusion element, C_f the film capacitance, and R_f the film resistance. Comparison between experimental EIS spectra (black squares) and fitted results (red circles) for (b) SPE, (c) SPE/rGO, (d) SPE/rGO/AuNPs, (e) SPE/rGO/AuNPs/Aptamer, and (f) SPE/rGO/AuNPs/Aptamer/MCH electrodes. The excellent agreement between the experimental and fitted data confirms the validity of the equivalent circuit model. The extracted charge-transfer resistance (R_{ct}) values are summarized in Table S1.

Table S1. Charge-transfer resistance (R_{ct}) values obtained from equivalent-circuit fitting of the EIS spectra for different electrodes.

Electrode	R_{ct}	
	Value (Ω)	Error (%)
SPE	3584.0	0.283
SPE/rGO	110.2	1.946
SPE/rGO/AuNps	22.6	1.088
SPE/rGO/AuNps/Aptamer	240.7	4.898
SPE/rGO/AuNps/Aptamer/MCH	219.4	5.213
